# Field epidemiology training programs contribute to COVID-19 preparedness and response globally

**DOI:** 10.1186/s12889-021-12422-z

**Published:** 2022-01-10

**Authors:** Audrey E. Hu, Robert Fontaine, Reina Turcios-Ruiz, Aisha A. Abedi, Seymour Williams, Angela Hilmers, Eni Njoh, Elizabeth Bell, Carl Reddy, Kashef Ijaz, Henry C. Baggett

**Affiliations:** 1 Division of Global Health Protection, U.S. Centers for Disease Control and Prevention, 1825 Century Blvd NE, Atlanta, GA USA; 2 Public Health - Seattle & King County, Seattle, WA USA; 3 Training Programs in Epidemiology and Public Health Interventions Network, The Task Force for Global Health, Atlanta, GA 30345 USA; 4 The Carter Center, Atlanta, GA USA

**Keywords:** Field epidemiology, COVID-19, Global health, Pandemic response, Surveillance, FETP

## Abstract

**Background:**

Field epidemiology training programs (FETPs) have trained field epidemiologists who strengthen global capacities for surveillance and response to public health threats. We describe how FETP residents and graduates have contributed to COVID-19 preparedness and response globally.

**Methods:**

We conducted a cross-sectional survey of FETPs between March 13 and April 15, 2020 to understand how FETP residents or graduates were contributing to COVID-19 response activities. The survey tool was structured around the eight Pillars of the World Health Organization’s (WHO) Strategic Preparedness and Response Plan for COVID-19. We used descriptive statistics to summarize quantitative results and content analysis for qualitative data.

**Results:**

Among 88 invited programs, 65 (74%) responded and indicated that FETP residents and graduates have engaged in the COVID-19 response across all six WHO regions. Response efforts focused on country-level coordination (98%), surveillance, rapid response teams, case investigations (97%), activities at points of entry (92%), and risk communication and community engagement (82%). Descriptions of FETP contributions to COVID-19 preparedness and response are categorized into seven main themes: conducting epidemiological activities, managing logistics and coordination, leading risk communication efforts, providing guidance, supporting surveillance activities, training and developing the workforce, and holding leadership positions.

**Conclusions:**

Our findings demonstrate the value of FETPs in responding to public health threats like COVID-19. This program provides critical assistance to countries' COVID-19 response efforts but also enhances epidemiologic workforce capacity, public health emergency infrastructure and helps ensure global health security as prescribed in the WHO’s International Health Regulations.

**Supplementary Information:**

The online version contains supplementary material available at 10.1186/s12889-021-12422-z.

## Background

The COVID-19 pandemic has demanded robust, evidence-based, and coordinated preparedness and response efforts at national, regional, and global levels. Effective responses to public health threats require a well-trained public health workforce, especially field epidemiologists [1]. The International Health Regulations (IHR, 2005) sets the minimum target of trained field epidemiologists at 1 per 200,000 population [2, 3].

Field epidemiology training programs (FETPs) train qualified field epidemiologists that build global surveillance and response capacity [4]. Since 1980, FETPs have trained more than 18,000 individuals in over 80 countries [5]. Programs apply a competencies-based, practicum approach for each of the three tiers of training: Frontline, Intermediate, and Advanced [6]. Training duration ranges from 2 years for Advanced level, 9 months for Intermediate, and 3 months for Frontline [7, 8]. Graduates of the Advanced and Intermediate tiers contribute to the IHR field epidemiologist target of 1 per 200,000 population, while Frontline graduates strengthen the foundational surveillance capacities that permit early disease detection and opportune response. Graduates and residents of all levels become adept at preparing for, detecting, and responding to outbreaks on small and large scales.

FETPs have supported preparation and response activities to important public health threats such as Zika and Ebola virus disease [9, 10]. Recent reports have featured FETP contributions to national and regional COVID-19 responses. In the Eastern Mediterranean Region, graduates of the Jordan, Sudan, and Yemen FETPs have supported country efforts to manage the COVID-19 pandemic [11, 12]. However, the nature and extent of FETP contributions have not been systematically documented at a global level. The objective of this study was to describe how the work of FETP residents and graduates has contributed to COVID-19 preparedness and response around the world, in order to understand the value of investments in field epidemiology workforce development.

## Methods

### Study design and data

Between March 13 and April 15, 2020, we conducted a cross-sectional survey of FETPs that were either members of the Training Programs in Epidemiology and Public Health Interventions Network (TEPHINET) or supported by the U.S. Centers for Disease Control and Prevention (CDC). We designed a nine-item questionnaire with quantitative and qualitative elements to capture FETP residents’ and graduates’ COVID-19-related activities, categorized according to the eight Pillars (P) of WHO’s Strategic Preparedness and Response Plan for COVID-19: P1) Country-level coordination, planning, and monitoring; P2) Risk communication and community engagement, P3) Surveillance, rapid response teams, and case investigation; P4) Points of entry (PoE); P5) National laboratories; P6) Infection prevention and control (IPC); P7) Case management; and P8) Operational support and logistics [13].

Programs were asked whether FETP residents or graduates were involved in activities of each of the eight Pillars, and to provide a narrative description of the activities they performed. Programs were not asked how many residents or graduates, or which FETP tier (Frontline, Intermediate, or Advanced) were involved. Program directors or administrators were invited to complete the survey using SurveyMonkey® (San Mateo, USA). The majority of online surveys were completed in English; a few programs provided responses in French, Portuguese, or Spanish. At least two attempts were made to reach nonresponding programs by email or phone, and the survey was administered to 11 additional programs via telephone interviews. Responses in other languages were translated verbatim into English. Data were exported into Microsoft Excel and MAXQDA Plus for analysis.

### Quantitative analysis

Using the survey data, we calculated the number and percentage of programs with FETP residents or graduates involved in COVID-19 preparedness or response activities by region and WHO Pillar. In addition, we summarized select characteristics of participating programs, including age of program, training tiers, and interval between date of country’s first COVID-19 case and survey completion, using descriptive statistics. Data regarding the age of program and training tiers were provided by TEPHINET [14], and the date of country’s first COVID-19 case was collated from WHO Situation Reports [15].

### Qualitative analysis

Four co-authors used content analysis to summarize the narrative descriptions of resident or graduate COVID-19-related activities. Each person developed initial codes independently to identify recurrent phrases describing COVID-19 related activities. The team met to discuss each code following the coding of the first few FETP responses and developed code descriptions to use for the remaining coding process. After completing the country-by-country review and developing more than 250 codes, the team reconciled and organized these codes into categories. The team split up the categories, and each author refined codes within those categories by referring back to the responses in a WHO Pillar-by-Pillar review. The refined codes were further discussed, and consensus was reached. The team identified themes across categories and developed theme descriptions [16].

## Results

### Quantitative

**Fig. 1:**
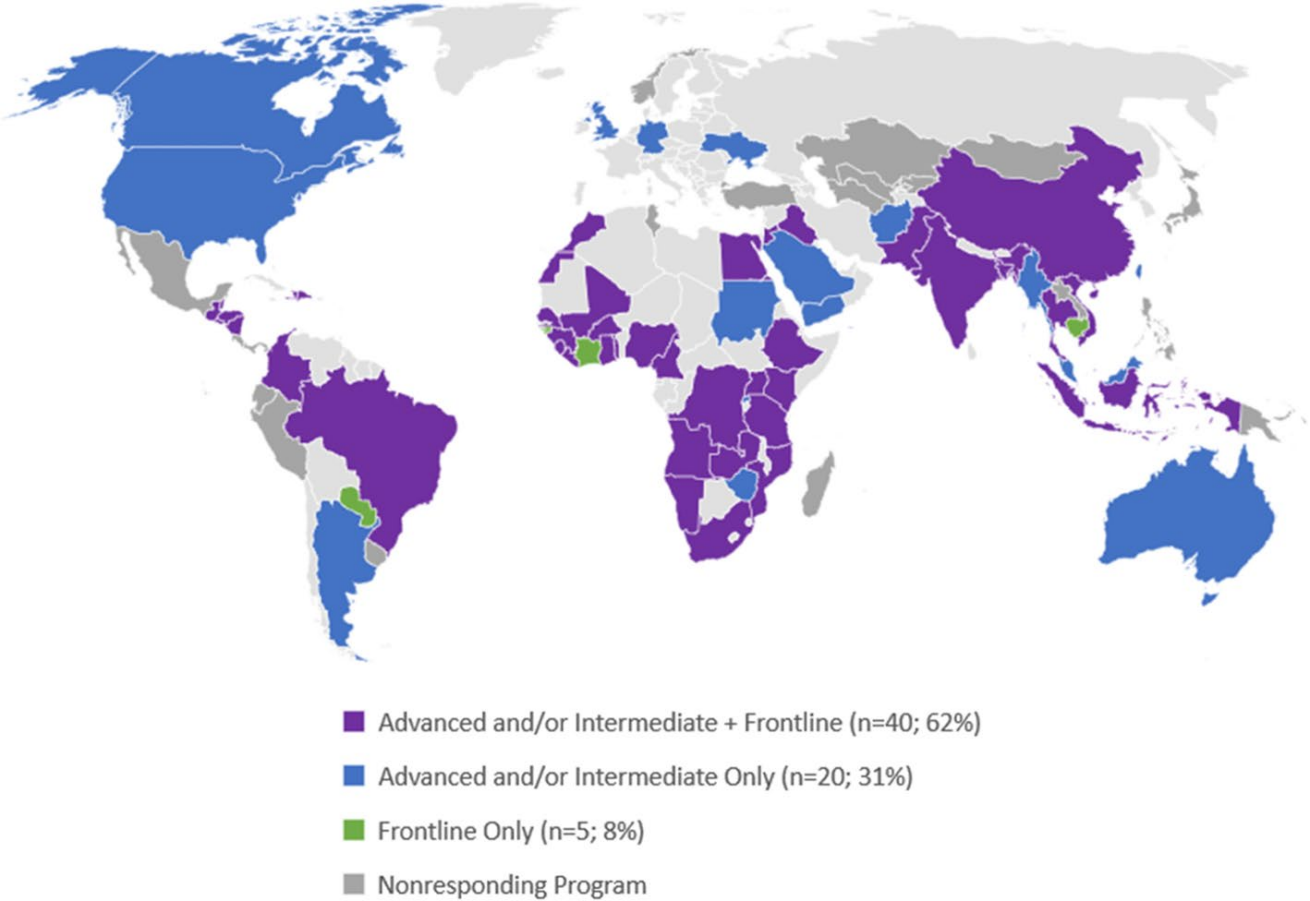
Geographic distribution and field epidemiology training tier available to countries served by the 65 Field epidemiology training programs that participated in the survey, March 13 – April 15, 2020. **Included in the 65 responding programs were the following regional programs, each of which serves multiple countries: Caribbean Regional FETP, Central America FETP, European Programme for Intervention Epidemiology Training (EPIET) and European Programme for Public Health Microbiology Training (EUPHEM), and South Caucasus FETP

Of 88 invited programs, 65 (74%) responded, encompassing all WHO Regions. Responding programs served 79 countries worldwide. Of these, 53 (82%) had an Advanced tier, 5 (7.7%) had Frontline only, and 41 (63%) had more than one FETP training tier (Fig. 1).

Responding FETPs have conducted training for a median of 11 years (range: 1–69 years) (Table 1). The oldest program was the U.S. Epidemic Intelligence Service, established in 1951, and the youngest program was the Afghanistan Field Epidemiology Training Program, established in 2019.

**Table 1:** Summarized characteristics of Field epidemiology training programs that responded to the online survey between March 13–April 15, 2020

WHO region	Program age in years, median (range)	Days since first COVID-19 Case was reported in Country^a^, median (range)
AFRO: Africa	8 (2–27)	19 (3–35)
EMRO: Eastern Mediterranean	10 (1–31)	33 (14–51)
EURO: Europe	11 (2–25)	47 (23–52)
WPRO: Western Pacific	18 (9–36)	73 (56–105)
PAHO: Americas	19 (3–69)	27 (11–74)
SEARO: Southeast Asia	19 (2–40)	34 (16–74)
All Programs	11 (1–69)	25 (3–105)

^a^Regional programs serving multiple countries and four national programs (Guinea-Bissau, Mozambique, Sierra Leone, and Yemen) that responded to the survey before the first COVID-19 case was reported in their country were not included in the calculation of median days since first COVID-19 case.

When the survey was administered, 93% (56/60) of programs had reported a COVID-19 case in their country. Five regional programs that serve multiple countries were excluded from this calculation. Of those programs with a reported case, they answered the survey a median of 25 days after the first reported case (range 3–105 days). The programs without reported cases responded a median of 5 days before the first reported case in the country.

**Fig. 2:**
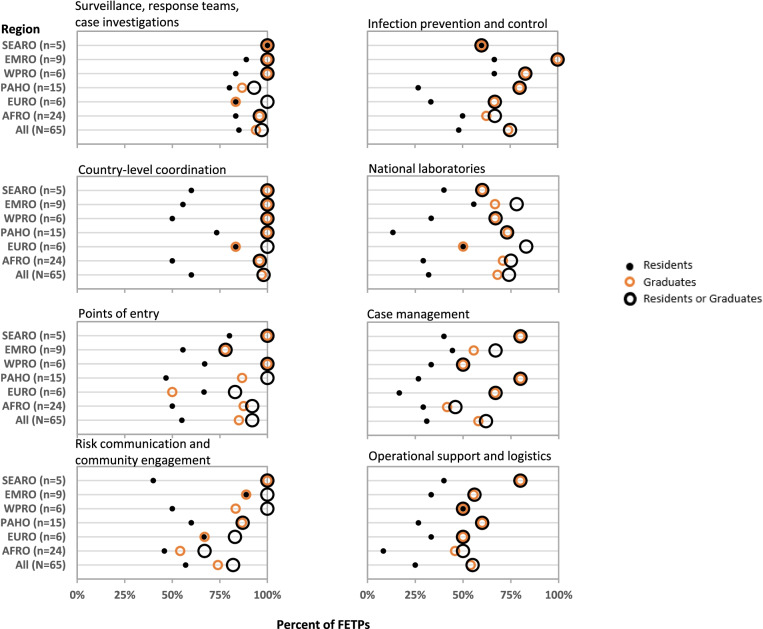
Frequency of Field epidemiology training programs (FETPs) reporting resident or graduate support to COVID-19 preparedness and response by WHO Preparedness and Response Pillars and WHO region. Programs indicating engagement of FETP residents, graduates, or any FETP involvement (residents or graduates) is shown. Preparedness and Response Pillars are sorted by mean response for all regions from the top left panel to the bottom right panel, and regions are sorted by mean response across the 8 Pillars

Survey respondents reported that FETP residents and graduates were engaged in the COVID-19 response in all six WHO regions and across all eight WHO Strategic Preparedness and Response Pillars (Fig. 2). COVID-19 response participation was uniformly high (median = 100%; range = 80–100%) in all regions for the following pillars: Surveillance, response teams, case investigation (P3), Country-level coordination (P1), and PoE (P4). These were followed closely by Risk communication and community engagement (P2). These WHO Pillars all involved competencies for which FETP was designed (Table 2). Engagement of FETPs in National laboratories (P5), IPC (P6), Case management (P7), and Operational support and logistics (P8) was reported at lower percentages (median = 67%; range = 46–100%) and with greater variability across WHO Pillars, among regions, and between residents and graduates. Across all WHO Pillars, FETPs used graduates more frequently in preparedness or response activities compared to residents. Countries most frequently used graduates in country-level coordination, while they used residents most frequently in surveillance, rapid response teams, and case investigations.

**Table 2:** Competencies in Field Epidemiology Training Programs by public health topic area [6]

Area	Competencies
Epidemiologic Methods	1. Use epidemiologic practices to conduct studies that improve public health program delivery 2. Respond to outbreaks
Biostatistics	3. Analyze epidemiologic data using appropriate statistical methods
Public Health Surveillance	4. Set up, manage, and evaluate a public health surveillance system
Laboratory and Biosafety	5. Use laboratory resources to support epidemiologic activities
Communication	6. Develop written public health communications 7. Develop and deliver oral public health communications
Computer Technology	8. Use computers for specific applications relevant to public health practices
Management and Leadership	9. Manage a field project 10. Manage staff and resources 11. Be an effective team leader and member 12. Manage personal responsibilities
Prevention Effectiveness	13. Apply simple tools for economic analysis
Teaching and Mentoring	14. Train public health professionals 15. Mentor public health professionals
Epidemiology of Priority Diseases and Injuries	16. Evaluate and prioritize the importance of diseases or conditions of national public health concern

### Qualitative

Seven themes emerged during content analysis that illustrate contributions of FETP to COVID-19 preparedness and response: epidemiologic activities, managing logistics and coordination, leading risk communication efforts, providing guidance, supporting surveillance activities, training and developing the workforce, and holding leadership positions (Table 3).

**Table 3:** Main themes identified from the qualitative responses provided by Field epidemiology training programs and their associated WHO Preparedness and Response Pillars

Main themes	WHO Pillars^a^
Conducting Epidemiological Activities	1, 2, 3, 4, 6, 7
Managing Logistics and Coordination	1, 2, 3, 4, 5, 6, 7, 8
Leading Risk Communication Efforts	1, 2, 3, 4, 5, 6, 7
Providing Guidance	1, 3, 4, 5, 6, 7
Supporting Surveillance Activities	1, 3, 4
Training and Developing the Workforce	1, 2, 3, 4, 5, 6, 7, 8
Holding Leadership Positions	1, 2, 3, 4, 5, 6, 7

Data source: 65 FETPs that responded to the online survey between March 13–April 15, 2020.

^a^Eight Pillars of WHO’s Strategic Preparedness and Response Plan for COVID-19:

1. Country-level coordination, planning, and monitoring.

2. Risk communication and community engagement.

3. Surveillance, rapid response teams, and case investigation.

4. Points of entry.

5. National laboratories.

6. Infection prevention and control.

7. Case management.

8. Operational support and logistics.

#### Conducting epidemiological activities

FETPs have led and supported a variety of field epidemiology activities such as contact tracing, case investigations, sample collection, and development of situation reports. “*When a case is identified…they confirm meeting case definition and call the laboratory for a lab team to collect samples. They identify contacts. The rapid response teams conduct interviews and trace contacts and follow-up on the isolation measures that should be taken, that all contacts of confirmed cases are in quarantine.*” (Angola) FETP residents and graduates have deployed to state, local, and subnational districts to support epidemiologic response efforts. “*Our graduates and residents are also actively engaged in state health departments conducting cluster investigations, analyzing data, developing situation reports.*” (Australia) “*Graduates who work as local epidemiologists at points of entry have conducted case detection, sample collection, coordination of patient transport, and case investigation to make the decision of whether and where to isolate (*e.g. *Hospital).*” (Dominican Republic).

#### Managing logistics and coordination

FETPs have managed the logistics and coordination of the COVID-19 response in their countries. They have directly supported operations at national and local levels. *“FETP-Advanced is engaged in the planning and coordinating actions from the first moment of the pandemic; providing direct support to the Director of Epidemiology in many of the response activities at the central and regional levels.”* (Paraguay).

FETPs also provided logistical and operational support through management of supply chains and coordinating staff deployments. They have supported the response coordination amongst different institutions within the country, as well as with other countries. “*FETP graduates have taken an active part in planning, preparing a contingency plan, and inter-institutional coordination of the response.”* (Dominican Republic).

#### Leading risk communication efforts

FETPs have led efforts to develop communication materials and conduct risk communication activities to spread awareness, combat misinformation, and prepare messaging for their respective countries. *“There is a Department of Communication in the Ministry, but they [FETP] provide opinions on the content of the message and disposition of images and brochures.”* (Angola).

FETPs have been active in numerous venues to engage their communities, including social media, radio, press interviews, public awareness campaigns, leaflets and posters, and call centers. *“FELTPs advocated for the establishment of the toll-free hotline for COVID-19 response. FELTPs took charge of manning the call centre, operating 24 h, for 3 weeks, before they were joined by other cadres.”* (Namibia) *“Most of our fellows [FETP residents] are in the frontline, introducing the public to the national epidemic, the risk assessment results, the update on outbreak investigations, and effective preventive measures…82 graduates were interviewed by national, provincial, prefecture social media, TV, newspaper.”* (China).

#### Providing guidance

FETPs have supported the development of guidance in critical response activities, particularly IPC and case management. Early in the response, PoEs such as airports and borders were key locations to mitigate spread and FETPs had a strong presence, *“… providing personnel preventive guidance for staff at PoE, making disinfection guidance for the PoE, developing preparedness protocol for imported or migrated cases,”* (China).

Many FETP graduates and residents are also health care professionals with experience working in clinical settings. Their knowledge of patient documentation in these settings make them an asset in standardizing reporting forms for case management for all levels of the health structure*. “FETP fellows [residents] participated in updating the national standard reporting forms for COVID-19 cases and their contacts which were used to collect, report, analyze case-based information of COVID-19.”* (Egypt).

#### Supporting surveillance activities

FETPs in all WHO regions were heavily engaged in COVID-19 surveillance activities in a variety of response settings. Reported activities include case-based reporting, active disease surveillance, event-based surveillance, surveillance at borders and quarantine sites, and analyzing surveillance data. Programs activated rapid response teams with FETP residents making up part of this team. One FETP reported daily supervision of frontline workers’ *“…active house-to-house surveillance…in the containment zone,”* and *“line listing of the family members and those having symptoms.”*(India) *“At the quarantine zones, the graduates conduct epidemiological surveillance of quarantine zones, monitoring, case study, searching for contacts in the field, processing information.”* (South Caucasus).

#### Training and developing the workforce

FETPs have trained and developed the capacity of COVID-19 response staff in activities related to their expected response tasks including case management, correct use of PPE, risk communication, and screening. In one program, FETP residents and graduates conducted a *“cascaded training on COVID-19 preparedness to all 14 regions and 35 districts.”* (Namibia). Other capacity building activities included training *“family physicians on case management and contact tracing”* (South Caucasus), *“health personnel on sample collection and transport”* (Guatemala), and “*educating staff at a hotel [a quarantine site] about infection control measures to be observed while attending to suspected cases there under institutional quarantine,”* (Uganda).

#### Holding leadership positions

FETP residents and graduates have held leadership positions in a variety of response settings. In one program, “the National Department of Laboratory is led by a resident and a graduate. They are responsible of [sic] the national lab where the activities of the national lab are planned and coordinated.” (Angola).

Other FETPs provided leadership through coordinating activities at Emergency Operations Centers, serving as subject matter experts in risk communication, providing IPC guidance, and directing travelers at PoE. *“The provincial FELTP Disease Surveillance and Response Units are designated as focal points for the COVID-19 response. All case investigation and surveillance activities are being spearheaded, conducted, and coordinated through [them].”* (Pakistan).

## Discussion

Our findings demonstrate the breadth of FETP resident and graduate contributions to the COVID-19 pandemic response, supporting the relevance of their field epidemiology training to response activities. The WHO Pillars and response activities in which residents and graduates participated match well with the core competencies of their training: surveillance and case investigation with the public health surveillance competency; coordination, screening at PoE, and risk communication and community engagement with the field investigation and epidemiologic methods competencies.

The consistent engagement of residents and graduates across all WHO Pillars in their country’s national response reflects strong program integration with public health institutions. The near universal involvement of FETP graduates in national-level coordination is expected, as they have likely gained experience and seniority since graduation. The level of engagement varied between regions, likely reflecting some differences in programs, such as more involvement with national laboratories in FETPs with a laboratory component in training or greater familiarity with IPC practices and case management for FETPs that recruit residents with clinical backgrounds. Nonetheless, the high level of FETP graduate engagement across all WHO Pillars indicates that governments have maintained these field epidemiologists in appropriate positions in public service with the respective MOHs, national public health institutes, and other agencies that support public health functions.

FETPs are designed to teach residents field investigation and public health surveillance skills for the relevant and common national priority diseases and conditions, as well as any major public health emergencies. Examples of public health emergencies that benefited from FETP responders include the Zika response in Puerto Rico and South America [17, 18], SARS in China [19–22], Ebola in West Africa and Democratic Republic of the Congo [9, 10], and environmental disasters such as the Indus River flooding throughout Pakistan in 2010 [4] and the 9-province ice storm in central China in 2008 [4]. The preparation of the FETP residents and graduates for handling these major emergencies is gained through years of mentored experiences while serving in localized and diverse public health responses. The COVID-19 pandemic represents an ultimate test of this development and investment in field epidemiology training.

Our findings support that national governments were familiar with FETPs and activated them early in the response, often for preparedness activities before detection of the first COVID-19 cases, such as screening and surveillance for COVID-19 at country borders, placing suspected cases in quarantine, assessing case importation risk and spread, and disseminating guidelines and information through media and hotlines. FETP involvement was most common within the four WHO Pillars most closely linked to core FETP competencies: Surveillance, response teams and case investigation (P3), Country level coordination (P1), PoE (P4), and Risk communication and community engagement (P2). FETP engagement within the remaining four WHO Pillars was lower but still over 50%. Notably, our survey was conducted relatively early in the response when a few countries had still not reported any COVID-19 cases. As the pandemic response needs have changed with increasing understanding of COVID-19 epidemiology and mitigation measures, FETP support has also changed over time.

The seven themes distilled from the qualitative analysis complemented the same four WHO Pillars that predominated in the quantitative analysis. These themes included the core competencies of FETP training including surveillance, epidemiologic investigation, outbreak response, and communication. Additionally, the qualitative analysis identified FETP contributions in areas that are not core parts of FETP training, such as managing logistics and coordination and provision of guidance. FETP engagement outside of core competency areas reflects the training-through-service orientation of these programs. FETPs are designed to position residents and graduates to serve countries’ priority public health needs. This service-oriented ethos promotes role flexibility that is often necessary during emergency responses.

In particular, the qualitative analysis highlighted the leadership, management, and training functions of FETPs in the COVID-19 response. Training and leadership were not listed as WHO Pillars but were nevertheless an important need of countries in their COVID-19 response. FETP residents have been recruited as trainers for local health units, surveillance workers, and other residents. Ideally, programs take on graduates as staff epidemiologists to train new cohorts, thus propagating this training function.

Interpretation of our findings is limited by several factors. Although we queried all 88 TEPHINET member FETPs and achieved a high (74%) response rate, non-responding programs may have differed from responding programs. Staff of non-responding programs may have been too busy with the COVID-19 response to reply or served in countries that had not yet recognized case importations at the time of survey administration. Non-TEPHINET member programs were also not represented. Our survey questions were framed according to a standardized set of WHO COVID-19 response pillars, but we did not provide the WHO definitions or a complete list of activities for each WHO Pillar. Differing responsiveness to open text fields and potential misinterpretation from the responder or the analyst may have added variability among programs in the qualitative analysis. Similarly, the final identification of themes to categorize response activities requires interpretation and is subject to the bias of the analysts. In addition, our unit of analysis was the program, so we were not able to quantify the number of individual FETP residents and graduates contributing to COVID-19 response activities. Knowing the numbers of individuals and the response activities they conducted would allow more in-depth description of the magnitude of FETP support. From the qualitative analysis and other anecdotal reports, we surmise that programs have assigned most of their residents and available graduates to COVID-19 response activities. Other information sources such as newsletters from field epidemiology networks like TEPHINET and various press releases about FETP suggest that FETPs have responded to COVID-19 in more ways than just the activities captured here [23, 24]. Finally, our findings rely on self-reported data from countries and regions with FETPs and do not include comparisons to countries without FETPs. Therefore, our study does not allow inference about the quality or impact of the COVID-19 response in countries with vs. without FETPs. Furthermore, countries’ disease burden, preparation and response to the pandemic, and public health resources as documented by Joint External Evaluations varied widely [25, 26], and so we did not find it appropriate to draw comparisons beyond the data we collected.

Our findings underscore the value of FETPs to build the field epidemiology workforce capacity that is so critical during responses to public health emergencies. Adequate field epidemiology workforce capacity takes time to develop and requires investment from national governments and donors as well as political commitment. FETPs have grown and evolved over decades and are recognized as a pathway to achieve IHR human resource capacities [3]. The recently developed Global Field Epidemiology Roadmap sets forth a vision for global field epidemiology capacity and charts a path to achieve this vision through FETP [27, 28].

## Supplementary Information

**Additional file 1: Supplemental Table.** Descriptions of main themes identified through qualitative analysis of survey responses.

## Data Availability

The datasets used and analyzed during the current study are available from the authors on reasonable request and with permission of the corresponding Field Epidemiology Training Program.
