# Supplementary material for: Field epidemiology training programs contribute to COVID-19 preparedness and response globally
Source: BMC Public Health. 2022 Jan 10;22:63. doi: 10.1186/s12889-021-12422-z (PMC8747444; doi:10.1186/s12889-021-12422-z)
Supplement: Supplementary file 1 — Additional file 1: Supplemental Table. Descriptions of main themes identified through qualitative analysis of survey responses. [file 12889_2021_12422_MOESM1_ESM.docx]

Field Epidemiology Training Programs contribute to COVID-19 preparedness and response globally

**Authors:** Audrey E. Hu MPH,^1^ Robert Fontaine MD,^1^ Reina Turcios-Ruiz MD,^1^ Aisha A. Abedi MPH,^1^ Seymour Williams MD,^1^ Angela Hilmers MD,^2^ Eni Njoh MPH^1^, Elizabeth Bell MPH,^1^ Carl Reddy, MBBCh,^2^ Kashef Ijaz MD^1*^, Henry C. Baggett MD^1*^

^1^ Division of Global Health Protection, U.S. Centers for Disease Control and Prevention, Atlanta, GA, USA

^2^ Training Programs in Epidemiology and Public Health Interventions Network, The Task Force for Global Health, Atlanta, GA, USA

*Both authors contributed equally in the senior author role

**Running Title:** Field Epidemiology Training Programs and COVID-19 Response

**Keywords:** field epidemiology, COVID-19, global health, pandemic response, surveillance

**Corresponding Author:** Henry C. Baggett, 1825 Century Blvd NE Atlanta, GA 30345. Phone: (404) 498-1224. Email: [hfb8@cdc.gov](mailto:hfb8@cdc.gov)

**Disclaimer:** The findings and conclusions in this study are those of the author(s) and do not necessarily represent the official position of the U.S. Centers for Disease Control and Prevention

**Supplemental Table:** Descriptions of main themes identified through qualitative analysis of survey responses

| Conducting Epidemiologic Activities | Managing Logistics and Coordination | Leading Risk Communication Efforts | Providing Guidance |
| --- | --- | --- | --- |
| FETPs have led and supported a variety of field epidemiology activities such as contact tracing, case investigations, sample collection, production of situation reports, etc. | FETPs have supported public health management of the COVID-19 response through coordination of activities, logistical support, operational support, and management of supply chain and deployments. | FETPs have led efforts to develop communication materials and conduct risk communication activities, to ensure information reaches their respective communities. | FETP have supported the development of guidance in critical response activities, particularly infection prevention and control, and case management. |
| Supporting Surveillance Activities | Training and Developing the Workforce | Holding Leadership Positions |  |
| FETPs were engaged in COVID-19 surveillance, engaging in case-based reporting, active disease surveillance, event-based surveillance, surveillance at borders and quarantine sites, and analyzing surveillance data. | FETPs have strengthened workforce capacity through training of response staff in COVID-19-relevant topics including infection prevention and control measures, correct use of PPE, contact tracing, sample collection and transport procedures, screening, and case management. | FETPs have provided critical leadership in a variety of settings: coordinating activities at the EOC, as SME in risk communication, providing infection prevention and control guidance, directing travelers at points of entry. |  |
